# Experimental Evolution of Interference Competition

**DOI:** 10.3389/fmicb.2021.613450

**Published:** 2021-03-25

**Authors:** Florien A. Gorter, Carolina Tabares-Mafla, Rees Kassen, Sijmen E. Schoustra

**Affiliations:** ^1^Laboratory of Genetics, Wageningen University & Research, Wageningen, Netherlands; ^2^Department of Environmental Systems Science, Eidgenössische Technische Hochschule, Zurich, Switzerland; ^3^Department of Environmental Microbiology, Eawag, Dübendorf, Switzerland; ^4^Department of Biology, University of Ottawa, Ottawa, ON, Canada

**Keywords:** interference competition, repeated serial transfer, *Pseudomonas aeruginosa*, bacteriophage, pyocins, bacteriocins, clinical isolates

## Abstract

The importance of interference competition, where individuals compete through antagonistic traits such as the production of toxins, has long been recognized by ecologists, yet understanding how these types of interactions evolve remains limited. Toxin production is thought to be beneficial when competing with a competitor. Here, we explore if antagonism can evolve by long-term selection of the toxin (pyocin) producing strain *Pseudomonas aeruginosa* PAO1 in the presence (or absence) of one of three clinical isolates of the same species (*Recipient*) over ten serial transfers. We find that inhibition decreases in the absence of a recipient. In the presence of a recipient, antagonism evolved to be different depending on the recipient used. Our study shows that the evolution of interference competition by toxins can decrease or increase, experimentally demonstrating the importance of this type of interaction for the evolution of species interactions.

## Introduction

Ecologists often distinguish between two types of competitive interaction. The first, exploitative competition, occurs through the depletion of a common resource: interactions among individuals are indirect, mediated solely through resource availability. The second is interference competition, where negative interactions occur directly through antagonistic traits such as the production of toxic chemicals, overgrowth, or aggressive behavior (Amarasekare, 2002; Hibbing et al., 2010). While the importance of both forms of competition in structuring natural communities has long been appreciated, our understanding of how such interactions, especially interference competition, evolve remains limited (Thompson, 1999; Kirkelund Hansen et al., 2007; Rendueles et al., 2015).

The evolution of interference competition between a focal strain that causes antagonism (the producer) and a competitor strain that is harmed (the recipient) involves a trade-off between the cost of producing the antagonistic trait and the benefits derived from eliminating competitors. Clearly interference competition should evolve only when the benefits outweigh the costs, however identifying the conditions under which this happens has proven challenging. Work on the production of antagonistic traits in bacteria like bacteriocins, proteinaceous toxins that specifically inhibit closely related (yet non-identical) competitors, has demonstrated a preferential benefit to the producer when growing in structured environments (Chao and Levin, 1981; Riley, 1993; Gordon and Riley, 1999; Riley and Gordon, 1999; Kerr et al., 2002; Riley and Wertz, 2002; Wloch-Salamon et al., 2008; Doekes et al., 2019). Under well-mixed conditions, by contrast, producers do not enjoy the same benefits of toxin production because the toxin is shared with all individuals, creating an opportunity for cheats who do not pay the cost of production but gain some or all of the benefits to flourish. Similar results have been obtained for other bacterial exotoxins (such as Le Gac and Doebeli, 2010).

The relative costs of production could further be modulated by the strength of resource competition. Where resource competition is absent because no competitors are present, there is no advantage to producing an antagonistic trait: a genotype that avoids producing the antagonistic trait should enjoy a fitness benefit and so antagonism should steadily degrade over time (Riley and Gordon, 1999; Vriezen et al., 2009). In the presence of competitors, the level of antagonism that evolves could depend on the extent to which the producer and competitor overlap in various aspects of ecology relevant to competition such as resource use. In general, high degrees of resource overlap, for example, could lead to increased antagonism, since eliminating competitors frees up additional resources. Lower levels of resource overlap would thus be expected to result in the evolution of decreased antagonism, since the cost of producing an antagonistic trait would not be offset by the benefits of extra resource availability (Doekes et al., 2019).

Here, we performed an exploratory study on the evolution of antagonism. We evolved a laboratory strain of the pathogenic bacterium *Pseudomonas aeruginosa* (PAO1) in a spatially structured environment for ten serial passages in the absence or presence of one of three non-evolving clinical “recipient” strains whose provenance is described in Aaron et al. (2010). PAO1 inhibits the growth of all three recipients, and differs in its relatedness to each of them (Schoustra et al., 2012), resulting in variations in the levels of antagonism and inhibition between the PAO1 “producer” strain and the various recipients. Previous work has shown that ecological dissimilarity, measured in terms of shared resource use, increases as genotypes become more distantly related (Schoustra et al., 2012). We used phenotypic assays to monitor levels of phenotypic change (of fitness/yield and inhibition). We expect that fitness will increase and that inhibition will evolve downwards (reduced production of inhibitory compounds) in the absence of any recipient. In the presence of a recipient, inhibition may evolve upwards or downwards. As we do not know the exact mechanism by which PAO1 inhibits each of the recipients, more specific predictions about the expected increase or decrease of toxin production/efficiency are difficult to make. Nevertheless, we can make some generic predictions about the mechanisms responsible: decreases in inhibition should be non-specific, being driven by loss-of-function mutations, whereas increases in inhibition are likely to be more specific, possibly gain-of-function, mutations targeting a particular recipient. To complement our phenotypic characterization, we sequenced selected evolved populations to assess changes at the genomic level.

## Materials and Methods

### Strains and Culture Conditions

Standard laboratory strain *Pseudomonas aeruginosa* PAO1 was used as the evolving strain (Stover et al., 2000; Klockgether et al., 2010). Three clinical *P. aeruginosa* isolates from cystic fibrosis patients (Aaron et al., 2010), were used as recipients: Pa07 = *Recipient 1*, Pa118 = *Recipient 2*, and Pa180 = *Recipient 3*. To create the ancestral evolving strain, a selectable tetracycline resistance marker and the *lac*Z gene were introduced into PAO1, using a protocol originally designed for *Pseudomonas fluorescens* (Jasmin and Kassen, 2007; Bailey and Kassen, 2012). On agar plates containing X-gal (5-bromo-4-chloro-3-indolyl-β-D-galactopyranoside; 40 mg/L), strains carrying the *lac*Z gene give rise to blue instead of white colonies. Using competition experiments with the original PAO1 strain and the PAO1 strain with lacZ inserted, we verified that this insertion had no effect on fitness. The recipient differ in their genetic similarity to PAO1 (see Supplementary Figure 1) (Schoustra et al., 2012), which previous work has allowed to correlate to ecological (dis)similarity. Recipients are resistant to nalidixic acid but sensitive to tetracycline, and do not carry the *lac*Z gene. PAO1 is sensitive to nalidixic acid. The tetracycline marker (in PAO1) and the nalidixic acid resistance marker was introduced by selecting a spontaneous resistant mutant from the original strain. Using competition experiments with the original recipient strains and the recipient strain with the antibiotic resistance marker, we verified that the presence of this marker had no effect on fitness. Unless otherwise specified, strains were grown in liquid lysogeny broth (LB) at 37°C for 24 h.

### Experimental Evolution Protocol

The ancestral evolving strain was cultured on semi-solid agar plates with or without a recipient for ten serial transfers, each with a 3-day growth cycle. Recipient, when present, were not propagated over multiple cycles. Rather, recipient strains were grown up from frozen stocks for every transfer and used to make semi-solid (0.7% agar) LB + X-gal overlays on solid (1.3% agar) LB + X-gal plates (100 μL recipient culture/5 mL overlay). For the *No recipient* treatment, semi-solid agar without bacterial culture was used. Three 5-μL spots of PAO1 culture were placed on top of each overlay, and three replicate plates were used per treatment, resulting in a total of 36 evolving lineages. The edge of the colony is clearly visible because PAO1 produces blue colonies on medium containing X-gal. After 3 days of incubation, the outside of each colony was sampled with a needle and transferred to 50 μL M9 minimal salts solution, collecting around 10^8^ cells (number verified using plate counts of a series of representative colonies). To initiate a new round of selection, 5 μL of each M9 suspension (containing a potentially mixed population of around 10^7^ cells) was transferred to a fresh plate with semi-solid overlay. Periodically, samples of transferred cultures were checked for absence of recipient using the selectable markers for PAO1 (tetracycline resistance and blue colonies on medium with Xgal) and the recipient (naladixic acid resistance), confirming that there was no (observed) carry-over of recipient strains during serial transfer. After seven transfers, one lineage from the *Recipient 3* treatment was lost. The number of mitotic generations per serial transfer can be calculated assuming exponential population growth in a fully mixed culture (resulting in around 8 generations per growth cycle) or by using the doubling time of the cells and assuming linear colony expansion on a solid surface (resulting in around 50 generations per growth cycle). The actual number of mitotic generations most likely lies between these estimates.

### Fitness (Yield) and Inhibition Assays

Yield of the ancestral strain and evolved populations was determined in triplicate under the same conditions as the selection experiment as a proxy for fitness. After incubation, colonies were cut out of the plate and vortexed in 15 mL M9 for at least 30 s. The number of colony forming units (CFU) was then estimated using serial dilutions and plate counts on LB + tetracycline. For each environment, relative fitness was calculated using the yield of the ancestral strain as a reference. Inhibition (killing efficiency) was estimated in triplicate for the ancestral strain and evolved populations using the dilution method (Jacob, 1954). Briefly, strains were grown up in liquid LB for 48 h. After centrifugation, supernatant was filtered (0.20 μM) and serially diluted in M9. Overlays with the recipient of interest were prepared in the same way as for the selection experiment. Dilution series of supernatant were spotted on top of the overlays and plates were incubated for 48 h. The inverse of the highest dilution of sterile supernatant giving rise to inhibition was defined as the inhibition score and used as a proxy for inhibition (Jacob, 1954; Schoustra et al., 2012). Relative killing was calculated by diving the inhibition score of the evolved population by the inhibition score of the ancestral PAO1 strain.

### Statistical Analysis

Yield and inhibition scores were log transformed prior to analysis and expressed relative to the mean yield/inhibition score of the ancestral strain in the same environment. To determine the effect of evolution treatment (four levels: *No recipient*, *Recipient 1*, *Recipient 2*, and *Recipient 3*), assay environment (four or three levels, respectively), and the interaction between these terms on fitness and killing, we used linear mixed effects models with plate and lineage within plate as random effects. Local adaptation to each social environment [own *vs.* other environment (i.e., with/without recipient), and own *vs.* other recipient] was assessed using models in which the ‘assay environment’ term was replaced with these newly defined factors. A linear mixed effects model (with plate, lineage within plate, and assay environment within lineage as random effects) was also used to evaluate the effect of genetic similarity (continuous variable, testing both the linear and the quadratic term. Genetic similarity under the *No recipient* treatment taken to be 0) on killing. To assess whether the observed changes in inhibition could explain the observed changes in fitness, we used correlation tests for each environment separately, fitting the model on the mean inhibition and fitness for each lineage in each recipient environment instead of on the raw data (to account for non-independence). In cases of multiple comparisons, we used sequential Bonferroni tests. All statistical analyses were performed in R version 3.4.1 (R Core Team, 2013).

### Whole Genome Sequencing

To determine which genomic changes that had occurred during our selection experiment, we isolated genomic DNA from: (1) the ancestral evolving strain, (2) the three populations from the *No recipient* treatment with the lowest relative inhibition efficiency (NO_R.4, NO_R.5, and NO_R.6), and (2) the three populations from the *Recipient 1* treatment with the highest relative inhibition score (R_1.1, R_1.3, and R_1.5). Genomic DNA was extracted from 1 mL overnight culture in rich medium according to the ‘DNA purification from gram-negative bacteria using the Gentra Puregene Yeast/Bact Kit’ protocol of QIAGEN^®^. The DNA concentration was determined with QuBit. Library (PCR-free short-insert library) construction and whole genome shotgun sequencing was performed at BGI Tech Solutions in Hong Kong using an Illumina HiSeq4000 platform and 150 bp short-read sequencing technology (PE150) with 100x coverage.

### SNP and Indel Analysis

We used the CLC Genomics Workbench 9.5.3 for all sequencing analyses. Reads were trimmed to remove adapter sequences and low-quality regions (*Q* < 30), and discarded if very short (<50 bp). Mapping to the reference *P. aeruginosa* PAO1 genome was performed using standard settings. Variant detection for the evolved populations was performed using the low frequency variant detector, again using standard settings, except that we only considered variants with a minimum frequency of 10%, and used a relative read direction filter of 5%. Variants were filtered against control reads, i.e., the mapping of the ancestral strain, and kept if fewer than three control reads supported the variant. Annotation of variants was performed using the *P. aeruginosa* PAO1 CDS (consensus coding sequence) track.

### Copy Number Variation Analysis

Visual inspection of the read mappings pointed to altered coverage of several regions in the *P. aeruginosa* PAO1 reference genome for both the ancestral strain and the evolved populations, and the CLC coverage analysis tool indicated that a substantial part of these differences in coverage were likely caused by real CNV events. Importantly, in the NO_R.4 population, we detected a region of increased coverage for which the reads appeared to belong to three different haplotypes. *De novo* assembly of these reads yielded three contigs, two of which did not map to the PAO1 reference genome, but instead mapped to two prophage regions in the *P. aeruginosa* LESB58 genome (Winstanley et al., 2009). Repeating all sequence analyses with the LESB58 genome as the reference revealed that all evolved populations contained one or more phage-like regions that were not present in the ancestral strain. Split read analysis was used to identify the locations of phage integrations. See Supplementary Material for details.

## Results

Experimental populations of PAO1 were evolved in the presence (or absence) of one of three non-evolving recipient strains over multiple rounds of propagation. Evolved populations were assayed for fitness and the capacity to inhibit the growth of recipient strains for all evolved populations relative to the ancestral PAO1 strain that founded the evolution experiment.

### Fitness

**FIGURE 1:**
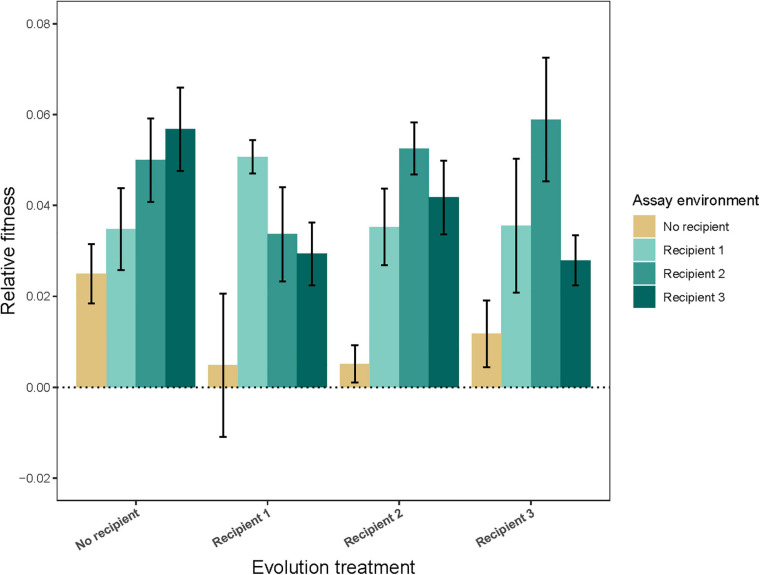
Relative fitness (±95% CI) of populations evolved in the absence (*No recipient*) or presence (*Recipient 1*, *Recipient 2*, or *Recipient 3*) of a non-evolving recipient strain, assessed in different assay environments. Bars show the average of nine independently evolved populations that were each assayed using three replicates.

Populations at the end of the experiment had increased yield, a proxy for fitness, relative to the ancestor in the environment in which they evolved (Figure 1; evolution treatment × assay environment: *F*_9_,_361_ = 5.03, *P* < 0.0001; fitness higher than 1 in all four cases; *P* ≤ 0.0015). Some degree of local adaptation also evolved. Specifically, strains evolved in the presence of Recipient *1* and *Recipient 2* performed better in the presence of their own *vs.* any other recipient. Conversely, strains evolved in the presence of *Recipient 3* performed worse in the presence of their own recipient (evolution treatment × own recipient *vs.* other recipient: *F*_2_,_196_ = 10.97, *P* < 0.0001; *P* ≤ 0.014 in all cases, see Supplementary Table 3). All strains, including the strains evolved in the absence of recipient, had a higher fitness in recipient environments than in no recipient environments (evolution treatment × own *vs.* other environment: *F*_3_,_361_ = 25.28, *P* < 0.0001; *P* ≤ 0.0001 in all cases), suggesting there has been adaptation to the conditions of culture and a general adaptation to the presence of a competitor (recipient).

### Inhibition

**FIGURE 2:**
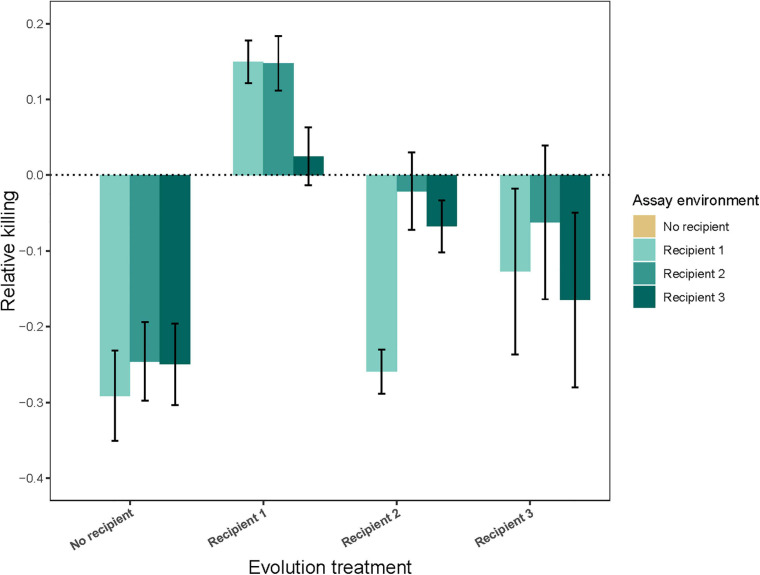
Inhibition (Relative killing ability ± 95% CIPA) of populations evolved in the absence (*No recipient*) or presence (*Recipient 1*, *Recipient 2*, or *Recipient 3*) of a non-evolving recipient strain, assessed in different assay environments (presence of either *Recipient 1*, *Recipient 2*, or *Recipient 3*). Bars show the average of nine independently evolved populations that were each assayed using three replicates.

The results for inhibition (killing efficiency) show that levels of inhibition have changed during our selection experiment, suggesting that our experimental protocol succeeded in selecting on antagonism. Populations from the *No recipient* treatment had a decreased inhibition relative to the ancestor in the presence of all three recipients (Figure 2; evolution treatment × assay environment: *F*_9_,_361_ = 24.28, *P* < 0.0001; inhibition score lower than 1 in all three cases *P* ≤ 0.0001; see Supplementary Table 4), and these decreases were not significantly different from each other after correcting for multiple comparisons. In the presence of their own recipient, strains evolved in the presence of *Recipient 1* had an increased inhibition, strains evolved in the presence of *Recipient 2* did not change in inhibition, and strains evolved in the presence of *Recipient 3* had a decreased inhibition (*Recipient 1*: *P* = 0.015, *Recipient 2*: *P* = 0.73, *Recipient 3*: *P* = 0.011). Strains evolved in the presence of *Recipient 1* and *Recipient 2* had a higher inhibition in the presence of their own *vs.* other recipient, while strains evolved in the presence of *Recipient 3* had a lower inhibition in the presence of their own *vs.* other *Recipient* (evolution treatment × own *Recipient vs.* other *Recipient*: *F*_2_,_205_ = 18.49, *P* < 0.0001; *P* ≤ 0.0091 in all cases).

To assess whether the observed changes in inhibition could explain the observed changes in fitness, we determined whether these two measures correlated in each of the three recipient environments. In the *Recipient 1* environment, inhibition and fitness were positively correlated, whereas in the *Recipient 2* and *Recipient 3* environment, these measures were negatively correlated. However, this correlation was significant only for the *Recipient 3* environment (*Recipient 1*: *r* = 0.17, *n* = 35, *P* = 0.33; *Recipient 2*: *r* = –0.18, *n* = 35, *P* = 0.32; *Recipient 3*: *r* = –0.36, *n* = 35, *P* = 0.038).

### Genomic Changes

**FIGURE 3:**
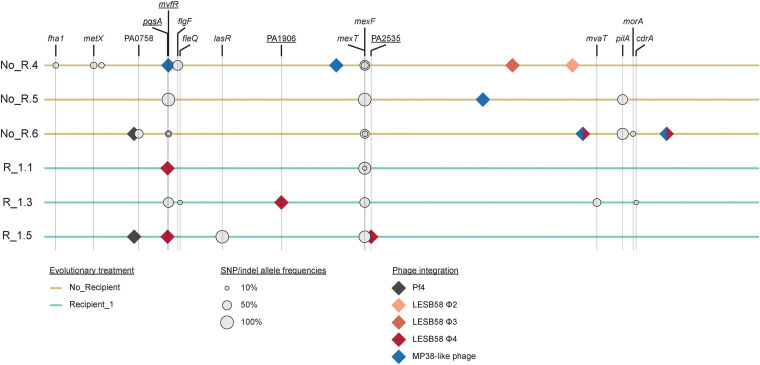
Genetic changes in populations evolved without recipient (No_R.4, No_R.5, and No_R.6) or in the presence of *Recipient 1* (R_1.1, R_1.3, and R_1.7). Relative size of gray circles reflects SNP or indel frequency within each population. All sequenced evolved populations contained one or more prophage elements that were not present in the ancestral strain. Genes that were disrupted by phage integration in at least one replicate population are underlined. Because border regions of LESB58 Φ4 and MP38-like phage are identical, it is not possible to determine which phage integrated where in population No_R.6 (two-colored diamonds). Supplementary Tables 1, 2 provide further details on specific SNPs and indels as well as on copy number variation.

We performed whole genome sequence analysis of the three populations from the *No recipient* treatment with the lowest inhibition (NO_R.4, NO_R.5, and NO_R.6), and the three populations from the *Recipient 1* treatment with the highest inhibition (R_1.1, R_1.3, and R_1.5). Our results revealed that on average 4.17 SNPs or small indels were segregating in each population at frequencies > 10%, and the mean number of such mutations per individual (i.e., the sum of the frequencies per population) was 1.83 (Figure 3 and Supplementary Table 1). These numbers were not significantly different between the two treatments (total number: *χ^2^* = 3.63, df = 1, *P* = 0.16, mean number: *t*_2_._86_ = 2.18, *P* = 0.12). The large majority of all mutations (22/25) conferred a non-synonymous change, and evolution was highly parallel at the gene and functional level. Three genes were hit independently multiple times (*mexT*: 7x, *mvfR*: 4x, and *pilA*: 2x), and most mutations were related—either directly or indirectly—to quorum sensing [or more specifically, to the Pseudomonas Quinolone Signaling (PQS) system; 16/25] or motility (pili or flagella; 4/25). With the exception of mutations in *pilA*, found exclusively in the *No recipient* populations, populations from different treatments did not differ substantially in terms of mutation identity.

In addition to the reported SNPs and indels, all evolved populations contained several prophage elements that were not present in the ancestral strain. Of these elements, Pf4 also occurs in the ancestral strain but an extra copy of this element is integrated at an additional genomic location in the evolved strains. LESB58 Φ2 – Φ4 are prophages that were first identified in the *P. aeruginosa* Liverpool Epidemic Strain (Winstanley et al., 2009), and are likely derived from the recipient strains. The same is potentially also true for the MP38-like prophage, which is highly similar to LESB58 Φ4. In one case, the MP38-like prophage integrated into a gene that was also hit by SNPs in other populations (i.e., in *mvfR* in population NO_R.4), and in a second case, the prophage integrated into the same gene in two independent populations (i.e., *pqsA* in populations E1 and E5). LESB58 Φ4 also integrated into two uncharacterized genes in populations E3 and E5 (see Supplementary Tables 1, 2 for full details).

## Discussion

We evolved a laboratory strain of the opportunistic pathogen *P. aeruginosa* in a spatially structured environment for 10 serial transfers in the absence or presence of one of three non-evolving sensitive competitor strains (“recipient”). While fitness (yield) increased in all environments, we found inhibition (killing) decreased in the absence of recipient. In the presence of recipient, inhibition showed a range of responses: it could decrease or increase, and be more or less specific to a particular recipient, depending on which recipient was used. Whole-genome sequencing of evolved populations revealed a range of possible mechanistic explanations for these results.

At the phenotypic level, we found that the direct response to selection, that is, the change in fitness among strains selected and assayed in their selection environment, was positive for all strains. This shows that selection was effective at causing adaptation over ten serial transfers, both in the presence and absence of competitor strains during selection. Interestingly, all strains, even those that evolved in the absence of Recipient, increased their performance when assayed in the presence of Recipient, likely due to generic adaptation to the laboratory selection environment. While selection in the absence of competitors did not compromise performance when assayed in the presence of competitors, the reverse was not true. Adaptation in the presence of competitors does not improve fitness to the same extent when competitors are absent, suggesting that adaptation in the presence of a recipient involves recipient-specific genetic effects. This interpretation is even more strongly reinforced when we consider evolutionary changes to the level of inhibition: prolonged evolution in the absence of recipient leads to a highest loss of antagonism. These results thus suggest that the social environment can impact the outcome of adaptive evolution and, in particular, that prolonged selection in the absence of competitors can lead to dramatic phenotypic changes involving the loss of antagonistic traits.

Theory suggests that prolonged selection to prevailing local conditions, whether they be abiotic or biotic, should lead to the evolution of specialization and local adaptation (Kaltz and Shykoff, 1998; Kassen, 2014; Schick et al., 2015). Assuming that this would apply to antagonistic traits, adaptation to a particular recipient in our experiment should then be specific, meaning that any gains in fitness or inhibition should be restricted to the recipient on which the producer evolved. Decrease in killing could be due to loss-of-function mechanisms and could be more generic to other recipients than the one present during selection. Our results provide mixed support for this idea. Inhibition evolved a degree of specificity on *Recipient 1* and *2*: inhibition of the ‘own’ strain was generally higher than for ‘other’ strains. There was no evidence for local adaptation in inhibition in *Recipient 3*, however. It is difficult to draw general conclusions from these results beyond the broad statement that the response to selection in the presence of un-evolving competitors can be highly variable and that the degree of inhibition is a trait that can evolve both upwards and downwards. The factors governing the extent to which the suite of antagonistic traits have general or specific effects on recipient remains a topic for further exploration. It may be that prolonged coevolution could lead to more specific adaptation, as has been observed previously in phage-bacteria experiments (Koskella and Brockhurst, 2014).

Interestingly, we observed that for at least one producer-recipient combination (*Recipient 1*) both inhibition and fitness increased in the producer strain. This result supports the notion that interference competition by means of anti-competitor toxins, while costly to the producer, can be associated with an increase in fitness. Moreover, it provides a direct demonstration that antagonistic traits could be linked to positive selection. A caveat to this interpretation is that we do not know precisely which of the range of toxins available to producers are under selection; chemical and other analysis that the inhibition we observed is due to these toxins is required (Oluyombo et al., 2019). At the same time, the lack of a strong correlation between inhibition and fitness in the other two producer-recipient combinations suggests that increases in inhibition are not the only way to evolve increased competitive ability. It is possible that adaptation via increased growth rates or swarming motility can lead to more effective resource capture without changes to antagonistic traits *per se*. Indeed, a previous study similar to ours found an evolutionary decrease in toxin-production against a sensitive competitor in both well-mixed and spatially structured environments following selection alongside the evolution of so-called defector genotypes that are resistant to but do not produce the toxin (Le Gac and Doebeli, 2010). Importantly, these results reinforce the notion that even when a sensitive competitor is present, the benefits of killing or inhibition do not always outweigh the costs, and recipient identity, as well as details of the experimental set-up, can play important roles in determining evolutionary outcomes.

When evolving in the presence of *Recipient*, we may expect the outcome of adaptation to depend on the degree of niche overlap or ecological dissimilarity, which here we measure using genetic similarity between producer and recipient as a proxy based on previous work (see Schoustra et al., 2012). This expectation is based on the idea that antagonism should evolve to be weak among ecologically distinct lineages because the producer and recipient strains have very different resource requirements and so do not, in practice, compete for the same resources (Kassen and Bell, 2000; Schoustra et al., 2012). Antagonism should also evolve to be weak among genetically (almost) identical strains, such as kin or offspring, because there is no fitness benefit to toxin production aimed at killing close relatives. At intermediate levels of genetic similarity, however, resource requirements are expected to be similar enough that it pays to produce a toxin to eliminate competitors, making the resources the competitor would have used available to the producer. Thus, selection for antagonism should be strongest when genetic similarity and associated resource requirements are intermediate (Gardner et al., 2004; Wloch-Salamon et al., 2008; Inglis et al., 2009; Le Gac and Doebeli, 2010; Schoustra et al., 2012). This interpretation is consistent with the view that microbial interference competition could be an important driver of biodiversity and virulence (Gardner et al., 2004; Rendueles et al., 2015). Because it can have often strong negative impacts on the reproductive success of the competitor, mechanisms mitigating the impacts of interference competition should readily evolve (Libberton et al., 2015; Inglis et al., 2016).

A direct test of this hypothesis requires tracking the fate of competing lineages that cover a wide range in genetic relatedness and ecological similarity over evolutionary time. In our work, only three recipients were used and thus do not provide a strong test of this idea. However, our results are consistent with the idea that antagonism should evolve to be strongest between strains that show intermediate levels of genetic relatedness. In the presence of *Recipient 1*, inhibition evolved upwards, in the presence of *Recipient* 2 inhibition remained constant and in the presence of *Recipient* 3 inhibition evolved downward. If we include the *No Recipient* environment in our analysis, we do find the expected quadratic relationship between evolved inhibition and genetic similarity (*F*_1_,_9_ = 7.62, *P* = 0.022; marginal *R*^2^ = 0.26; maximum inhibition ability predicted at 0.445 genetic similarity; see Supplementary Figure 1 and Supplementary Table 5), however with only four points this is not a very strong or compelling test of the hypothesis. Nonetheless, very few experiments to explore this theoretical prediction exist, and our results clearly warrant further investigation. Apart from using a wider range of *P. aeruginosa* strains as recipients, related species such as *P. putida* and *P. fluorescens* could be included to further broaden the range of relatedness between toxin producer and recipient strains.

To assess evolutionary changes at the genomic level, we performed whole genome sequencing of selected populations that had showed the highest increase or decrease in inhibition. A more detailed discussion on the genomic changes we observed is provided in the Supplement and in Supplementary Tables 1 and 2. In contrast to our expectations, differences in inhibition were not obviously mediated by changes in bacteriocin production. Instead, strains with both decreased and increased inhibition had—in addition to multiple mutations that were shared at the gene level such as *mexT* and *mvfR* —acquired novel prophage elements (Figure 3). This result suggests that subtle differences in prophage identity and/or epistatic interactions between selected genetic changes interacted with the pre-existing interference competition arsenal to alter the level of killing and/or to promote fitness of evolving strains in general in the selective environment (See Supplementary Text 1). The source of these elements is most likely the clinically isolated recipient strains or from other clinical strains that were being cultured in the laboratory in experiments for other projects (Schoustra et al., 2012; Dettman et al., 2013; Dettman and Kassen, 2020). In case of the strains from the *No recipient* treatment, phage particles may have been transmitted via aerosols (Verreault et al., 2010; Harms et al., 2017). LES prophages are widespread among cystic fibrosis isolates (Knezevic et al., 2015), including the ones in used our laboratory (Dettman et al., 2013), and have previously been shown to provide a strong competitive advantage *in vivo* (Winstanley et al., 2009; Davies et al., 2016). MP38-like phage is highly similar to LESB58 Φ4 and may thus play a comparable role in clinical settings. Various parallel changes occurred (Figure 3). Follow-up work could focus on sequencing individual isolates from populations and could involve validations of gene knock out strains and/or allelic replacement to further characterize effects of individual mutations with respect to killing. This would also show if mutations found are shared by all strains within an evolved population or whether evolved populations have diversified with respect to their genetic changes.

The advantage of harboring lysogenic prophages is potentially threefold. First, it directly confers resistance to superinfection by the same and closely related phages via the modification of surface receptors (type IV pilus and/or LPS receptors, Bondy-Denomy et al., 2016). Second, loss of surface piliation/twitching motility increases dispersal ability and thus fitness under our experimental conditions (i.e., on semi-solid agar where the edge of the colony is transferred, Taylor and Buckling, 2010). Such increases in dispersal ability could potentially explain the fitness advantage associated with prophage acquisition in the *No Recipient* treatment. Finally, lysogeny provides a direct competitive advantage in the presence of non-lysogenized clone mates, thus allowing lysogens to increase in frequency within their own population (Brown et al., 2006). While the first advantage is unique to the *Recipient* environment, the second and third may explain the increase in lysogen frequency in the absence of recipient. Even if lysogeny does not alter inhibition ability *per se* (the Recipient should already be resistant to the phage), it may interact with the existing arsenal of other prophages and bacteriocins in the evolving strain to increase their efficacy (Nedialkova et al., 2016).

Our results show that the level of interference competition can readily evolve to be more or less antagonistic. This has potentially important implications for pathogen evolution *in vivo*, because the level of interference competition has previously been shown to be inversely correlated with virulence (Massey et al., 2004; Inglis et al., 2009). Moreover, interference competition contributes to positively frequency-dependent interactions, and as such can drive the maintenance of biodiversity in spatially structured environments (Czárán et al., 2002; Rendueles et al., 2015; Wright and Vetsigian, 2016). Our work also suggests that exchange and recombination of “modular” interference competition elements may play a more important role in adaptation than fine-tuning of the existing interference competition arsenal, at least in the short term. This highlights the importance of mobile genetic elements for rapid adaptation (Hall et al., 2017), particularly in environments where organisms invade a resident population from rare (Brown et al., 2006). The genetic architecture of our particular model system plays a no doubt crucial role in our observations (Oluyombo et al., 2019). Nonetheless, this finding indicates that interference competition dynamics may not be fully captured by the commonly used theoretical models that view this process as a game of rock-paper-scissors (Czárán et al., 2002; Kerr et al., 2002; Gardner et al., 2004; Biernaskie et al., 2013), implying that such models will need to be extended if they are to more accurately capture real-world interference competition dynamics.

## Data Availability Statement

The data presented in the study are deposited in the NCBI database repository, accession number PRJNA707144. https://www.ncbi.nlm.nih.gov/sra/PRJNA707144.

## Author Contributions

FG, RK, and SS conceived (parts of) the study. SS and CT-M carried out the laboratory selection experiments. CT-M performed the DNA extractions for sequencing. FG and SS performed the data analysis. FG made the figures. FG, RK, and SS drafted the manuscript with input by CT-M. All the authors contributed to the article and approved the submitted version.

## Conflict of Interest

The authors declare that the research was conducted in the absence of any commercial or financial relationships that could be construed as a potential conflict of interest.

## Supplementary Material

Supplementary Text 1 Background and discussion on genomic changes that occurred in evolved populations.

Supplementary Table 1 SNPs and small indels in evolved populations.

Supplementary Table 2 Copy number variants in evolved populations.

Supplementary Table 3 Model output of the linear mixed model for changes in relative fitness.

Supplementary Table 4 Model output of the linear mixed model for changes in relative inhibition.

Supplementary Table 5 Model output of the linear mixed model for the effect of genetic similarity.
