# Supplementary material for: Experimental Evolution of Interference Competition
Source: Front Microbiol. 2021 Mar 25;12:613450. doi: 10.3389/fmicb.2021.613450 (PMC8027309; doi:10.3389/fmicb.2021.613450)
Supplement: Supplementary Text 1 — Background and discussion on genomic changes that occurred in evolved populations. [file Data_Sheet_1.docx]

Experimental evolution of interference competition

Florien A. Gorter, Carolina Tabares-Mafla, Rees Kassen, and Sijmen E. Schoustra

SUPPLEMENTARY INFORMATION

Main article: Front. Microbiol. 12:613450. doi: 10.3389/fmicb.2021.613450

**Supplementary Text 1.**

**Background and discussion on genomic changes that occurred in evolved populations**

**Pseudomonas Quinolone Signal** **PQS.** All sequenced evolved populations contained mutations in genes that either directly or indirectly affect the Pseudomonas Quinolone Signal (PQS). PQS regulates the expression of a wide range of virulence genes via the *pqsE* effector (Lee and Zhang, 2015; Rampioni et al., 2016; Baker et al., 2017), and can—depending on whether it is bound to iron—act as both an anti- and a pro-oxidant (Häussler and Becker, 2008). In this latter capacity, it induces the SOS response and associated downstream genes involved in DNA repair, bacteriocin production, cell lysis, and prophage activation (Argenio et al., 2002; Penterman et al., 2014). *mexT* is a mutational hotspot and a positive regulator of the mexEF-oprN efflux pump that exports the PQS precursor HHQ (Lamarche and Déziel, 2011). Loss-of-function of this gene increases internal PQS levels and elicits global phenotypic changes such as enhanced growth in early exponential phase, increased swarming motility, and altered resistance to several genotoxic agents (Luong et al., 2014; Penterman et al., 2014). By contrast, loss-of-function of *mvfR*, *pqsA*, *lasR,* and *mvaT* decreases PQS levels (Déziel et al., 2005; Westfall et al., 2006; Rampioni et al., 2016), although the precise effect of such mutations may vary (Mund et al., 2017). Interestingly, mutations in the first three of these genes were also selected repeatedly in a previous study when *P. aeruginosa* was grown in the presence of LESB58 Φ2 – Φ4, but not when it was grown in the absence of these phages (Davies et al., 2016). Taken together, these observations suggest that mutations in *mexT* may have been selected initially for their positive effect on competitive ability, but that these mutations also induced prophage (in the evolving as well as the recipient strains) as a pleiotropic effect and transiently decreased resistance to phage-like compounds (i.e. phage and phage-like bacteriocins). Mutations that decrease PQS levels may then have been selected to compensate for these effects, i.e. to reduce the frequency of prophage-driven autolysis and to increase resistance to phage-like compounds.

**Prophage elements and parallel changes.** Interestingly, strains that had evolved in both the absence and presence of *Recipient* contained one or more novel prophage elements. The source of these elements is most likely the clinically isolated recipient strains. In case of the strains from the *No recipient* treatment, phage particles may have been transmitted via aerosols (Harms et al., 2017). LES prophages are widespread among cystic fibrosis isolates (Knezevic et al., 2015), and have previously been shown to provide a strong competitive advantage *in vivo* (Winstanley et al., 2009; Davies et al., 2016). MP38-like phage is highly similar to LESB58 Φ4 and may thus play a comparable role in clinical settings. The *fha1* mutation observed in one of the No_R evolved strains (See Supplementary Table 1) could indicate a role of the T6SS system, which could also be linked to phages due to its similarity in structure and is known to be part of delivering toxins to competitors (Smith et al., 2006; Chen et al., 2015).

The fact that populations from the *No recipient* and *Recipient 1* treatment contain SNPs and indels in the same genes suggests that the acquired prophages are responsible for the observed differences in inhibition between populations from these treatments. The difference in inhibition ability between *No recipient* and *Recipient 1* strains is potentially due to the genetic differences between LESB58 Φ4 and the MP38-like phage, the former being more prevalent in *Recipient 1* populations than the latter. Previously, such subtle genetic differences among prophage elements were shown to be responsible for *de novo* evolved interference competition in a pair of coevolving *Bacillus subtilis* strains (Martin et al., 2017). The advantage of harbouring lysogenic prophages is potentially threefold. First, it directly confers resistance to superinfection by the same and closely related phages via the modification of surface receptors (type IV pilus and/or LPS receptors (Bondy-Denomy et al., 2016)). Second, loss of surface piliation/twitching motility increases dispersal ability and thus fitness under our experimental conditions (i.e. on semi-solid agar where the edge of the colony is transferred (Taylor and Buckling, 2010). Such increases in dispersal ability could potentially explain the fitness advantage associated with prophage acquisition in the *No Recipient* treatment. Finally, lysogeny provides a direct competitive advantage in the presence of non-lysogenized clone mates, thus allowing lysogens to increase in frequency within their own population (Brown et al., 2006). While the first advantage is unique to the *Recipient* environment, the second and third may explain the increase in lysogen frequency in the absence of recipient. Even if lysogeny does not alter inhibition ability *per se* (the Recipient should already be resistant to the phage), it may interact with the existing arsenal of other prophages and bacteriocins in the evolving strain to increase their efficacy (Nedialkova et al., 2016).

**Regulatory genes.** We expected to find mutations in genes associated to production and regulation of pyocin (i.e. *P. aeruginosa* bacteriocin(Oluyombo et al., 2019), but did not observe this. In a way, the acquired prophage elements are not that different from R- and F-type pyocins, which are degenerate phage tails that are released via lysis of a subpopulation of all cells (Michel-Briand and Baysse, 2002). In fact, non-phage bacteriocins frequently coopt phage lysis genes for release into the environment (Nedialkova et al., 2016), and phages may themselves encode toxins (Jamet et al., 2017). This is maybe not surprising given that toxin production, immunity and lysis are genetically often closely linked: for example, R- and F-type pyocins, as well as the associated lysis genes, are all under the control of the same *prtR* and *prtN* regulators (Michel-Briand and Baysse, 2002), and although the exact mechanism of immunity is unknown, it seems likely that this trait is also encoded by a phage tail gene (Penterman et al., 2014; Bondy-Denomy et al., 2016). Increase in toxin production may thus be difficult to evolve without concomitant increase in lysis, and decrease in toxin production may be difficult to evolve without concomitant decrease in immunity (which is detrimental in the presence of wild-type clone mates (Inglis et al., 2013)). Indeed, in a large-scale temporal survey of clinical *P. aeruginosa* isolates, no mutations were observed in either *prtR* or *prtN*, and despite the fact that strains from later time points contained less pyocin genes on average, individual strains did not appear to have lost pyocin genes (Ghoul et al., 2015). Similarly, in a study with colicinogenic *E. coli*, strains that had evolved decreased inhibition ability contained no mutations in colicin genes, but instead had altered expression of DNA-repair and prophage genes (Vriezen et al., 2009).

Future work here could include a screening of what pyocins each of the evolved strains (and the ancestor) produce and what pyocins the recipient strains are sensitive to. These assays are not easy to do, yet novel antibody based techniques and the use of knock-out mutants may allow this. These assays could help fine-tune predictions on what structural or regulatory pyocins genes may be targets of selection.

**Copy number variation analysis.** Visual inspection of the read mappings pointed to altered coverage of several regions of the *P. aeruginosa* PAO1 reference genome for both the ancestral strain and the evolved populations. We used the CLC coverage analysis tool to detect regions where coverage was significantly different from the rest of the genome, and manually inspected reads tracks to confirm copy number changes, moving from the most significant regions until the point where we detected three or more apparent false positives in a row. Coverage data per position was then exported and analysed in R to calculate (relative) coverage for each region. This analysis revealed several real CNV events that were shared between the ancestor and the evolved populations. The most notable of these events is the ~2x coverage of the Pf4 prophage region (PA0718-PA0727; Supplementary table 2). Reads from this region contained several SNPs at intermediate frequency (mean frequency of SNPs at the—evenly—covered PA0720 locus: 51%), and unaligned ends of reads mapping to the borders of this region had 100% similarity with the *P. aeruginosa* PAO1 substrain MPAO1 phage-like insertion RGP42 genomic sequence (NCBI nucleotide BLAST). In combination with several other smaller CNV events and SNPs that we detected (i.a. two duplications at positions 5242058-5242171 and 5242935-5243029), this observation suggests that our ancestral strain was of the MPAO1 subtype, which contains, amongst other things, an RGP42 genomic island that is not present in the PAO1 reference strain (Klockgether et al., 2010).

In two of our evolved populations (NO_R.6 and R_1.5), coverage of the (extended) Pf4 prophage region was increased even further (Supplementary table 2). As above, we used the location of an accompanying amplification (a feature that is typically associated with the integration of genomic islands in *P. aeruginosa* (Klockgether et al., 2010)), the relative frequency of SNPs at the PA0720 locus, and the mapping of unaligned ends from the borders of this region, to elucidate the exact nature of these amplification events.

In addition to the above changes, we detected a relatively short region of increased coverage in the NO_R.4 population (673233-673489). The reads that mapped to this region appeared to belong to three different haplotypes, which led us to perform *de novo* assembly (increasing “word size” to 30) on these reads. This yielded three different contigs, two of which did not map to the PAO1 reference genome. Instead, BLASTing indicated that these contigs mapped in their entirety to the *P. aeruginosa* LESB58 genome sequence (Winstanley et al., 2009).

To determine whether our evolved population(s) had indeed acquired LESB58(-like) DNA, we repeated all sequencing analyses with the LESB58 genome as the reference. This revealed that all evolved populations contained DNA that was not present in the ancestral strain, and that (more or less closely) resembled DNA from one or more of three LESB58 prophage regions. Specifically, evolved population NO_R.4 contained DNA that was very similar to prophage regions 2 and 3, and populations NO_R.6, R_1.1, R_1.3, and R_1.5 contained DNA that was very similar to prophage region 4 (i.e. complete coverage, only a small number of SNPs). For populations NO_R.4, NO_R.5, and NO_R.6, reads mapped to the prophage 4 region, but with a large number of mismatches/gaps (for NO_R.6 this was the case for only part of the reads). To identify the — putatively prophage-like — genetic element responsible for this pattern, we performed *de novo* assembly on the NO_R.5 reads mapping to the prophage 4 region, as well as on all NO_R.5 reads. The largest contig from the prophage 4 region was then used to find the — larger — contig from the whole genome assembly that contained the putative prophage-like element. BLASTing revealed high similarity of part of this contig (96% over a length of 22944 bp) to bacteriophage MP38, which we interpreted as these populations having been lysogenized with an MP38-like phage.

To determine the mean copy number of the integrated phages in each population, we compared the coverage of each prophage region in the LESB58 genome (phages Φ2-Φ4) or the BLES_RS08060 gene (MP38-like phage; this gene is internal to the prophage 4 region, and is covered evenly with a limited number of mismatches in the MP38-lysogenized populations) to coverage of the PAO1 genome. For population NO_R.6, which contained both Φ4 and MP38-like phage, we used mean SNP frequency at the BLES_RS08060 gene to determine the relative amount of each phage. To detect where each phage had integrated into the genome, we extracted the reads that mapped to the borders of the LESB58 prophage regions (+/- 100 bp), and mapped them to the PAO1 reference sequence. We defined each location in the PAO1 reference where three or more split reads mapped uniquely as an integration site, and, in case of multiple integration sites within a single population, used the relative number of reads mapping to each site as a proxy for the relative amount of phage integrated at each location.

**Supplementary table 1**. SNPs and small indels in evolved populations.

| Line | Start position | Type | Reference | Allele | Frequency | Coding region change | Amino acid change | Gene | Annotation | Process/ component |
| --- | --- | --- | --- | --- | --- | --- | --- | --- | --- | --- |
| NO_R.4 | 98902 | Deletion | TGGCTG | - | 19.13 | 850_855delCAG CCA | Gln284_Pro285 del | PA0081 (*fha1*) | Fha domain-containing protein | type VI secretion system |
| NO_R.4 | 431534 | SNV | C | T | 25.42 | 236G>A | Trp79* | PA0390 *(metX)* | homoserine O-acetyltransferase | methionine biosynthesis |
| NO_R.4 | 499911 | Insertion | - | T | 19.12 |  |  |  | intergenic PA0444/PA0445 |  |
| NO_R.4 | 1168095 | Deletion | TCCTGG | - | 58.65 | 608_613delTCC TGG | Phe203_Glu205delins* | PA1081 (*flgF*) | flagellar basal-body rod protein | motility |
| NO_R.4 | 2807905 | SNV | G | A | 29.21 | 437G>A | Arg146His | PA2492 (*mexT)* | transcriptional regulator | quorum sensing |
| NO_R.4 | 2808673 | SNV | T | C | 59.19 |  |  |  | intergenic *mexT*/*mexE* | quorum sensing |
| NO_R.5 | 1086569 | SNV | T | G | 97.83 | 527A>C | His176Pro | PA1003 (*mvfR*) | transcriptional regulator | quorum sensing |
| NO_R.5 | 2807797 | SNV | C | A | 97.75 | 329C>A | Ala110Glu | PA2492 (*mexT)* | transcriptional regulator | quorum sensing |
| NO_R.5 | 5069225 | SNV | C | T | 59.93 | 307G>A | Asp103Asn | PA4525 (*pilA*) | type 4 fimbrial precursor | motility |
| NO_R.6 | 826621 | SNV | T | G | 45.16 | 679A>C | Thr227Pro | PA0758 | hypothetical protein |  |
| NO_R.6 | 1086890 | SNV | G | A | 24.68 | 206C>T | Pro69Leu | PA1003 (*mvfR*) | transcriptional regulator | quorum sensing |
| NO_R.6 | 1087093 | SNV | C | A | 10.77 | 3G>T | Met1? | PA1003 (*mvfR*) | transcriptional regulator | quorum sensing |
| NO_R.6 | 2807905 | SNV | G | A | 47.87 | 437G>A | Arg146His | PA2492 (*mexT)* | transcriptional regulator | quorum sensing |
| NO_R.6 | 2810330 | SNV | C | T | 18.90 | 322C>T | Gln108* | PA2494 (*mexF*) | multidrug efflux membrane fusion protein | quorum sensing |
| NO_R.6 | 5069240 | SNV | C | T | 75.37 | 292G>A | Asp98Asn | PA4525 (*pilA*) | type 4 fimbrial precursor | motility |
| NO_R.6 | 5160328 | SNV | T | G | 15.71 | 3961T>G | Tyr1321Asp | PA4601 (*morA*) | motility regulator | motility |
| R_1.1 | 2807797 | SNV | C | A | 87.60 | 329C>A | Ala110Glu | PA2492 (*mexT)* | transcriptional regulator | quorum sensing |
| R_1.1 | 2807905 | SNV | G | A | 11.34 | 437G>A | Arg146His | PA2492 (*mexT)* | transcriptional regulator | quorum sensing |
| R_1.3 | 1087034 | SNV | A | G | 59.36 | 62T>C | Ile21Thr | PA1003 (*mvfR*) | transcriptional regulator | quorum sensing |
| R_1.3 | 1188725 | SNV | T | G | 12.12 | 1139T>G | Val380Gly | PA1097 (*fleQ*) | transcriptional regulator | motility |
| R_1.3 | 2807797 | SNV | C | A | 57.85 | 329C>A | Ala110Glu | PA2492 (*mexT)* | transcriptional regulator | quorum sensing |
| R_1.3 | 4843819 | SNV | T | A | 37.40 | 8T>A | Leu3Gln | PA4315 (*mvaT*) | transcriptional regulator | quorum sensing |
| R_1.3 | 5188001 | SNV | A | G | 11.86 | 4875T>C |  | PA4625 (*cdrA*) | c-di-GMP-regulated TPS partner A | biofilm formation |
| R_1.5 | 1558397 | Deletion | TCAGTCACTGT | - | 96.30 | 227_237delTCA GTCACTGT | Val76fs | PA1430 (*lasR*) | transcriptional regulator | quorum sensing |
| R_1.5 | 2808007 | Insertion | - | CTAT | 80.49 | 539_540ins CTAT | Leu183fs | PA2492 (*mexT)* | transcriptional regulator | quorum sensing |

**Supplementary table 2**. Copy number variants in evolved populations.

| **Strain** | **Region** | **Genome** | **Mean coverage** | **Coverage relative to PAO1** | **Identity/source** | **Frequency prophage species** | **Integration site in PAO1 genome** | **Integration into gene** | **Frequency integration site** |
| --- | --- | --- | --- | --- | --- | --- | --- | --- | --- |
| ancestor | 789150..795786 | PAO1 | 940 | 1.99 | Pf4 (normal) | 49% |  |  |  |
|  |  |  |  |  | MPAO1 phage-like insertion RGP42 genomic sequence | 51% | 5243029..5242058 |  |  |
|  | 4788813..4789348 | PAO1 | 167 | 0.35 | deletion |  |  |  |  |
|  | 5253652..5254729 | PAO1 | 24 | 0.05 | deletion (same as in MPAO1) |  |  |  |  |
| NO_R.4 | 863878..906012 | LESB58 | 314 | 1.20 | LESB58 prophage 2 |  | 4629235..4629244 |  |  |
|  | 1433797..1476544 | LESB58 | 243 | 0.93 | LESB58 prophage 3 |  | 4103723..4103767 |  |  |
|  | 1683961..1720917 | LESB58 | 589 | 2.26 | MP38-like phage |  | 1086355..1086348 | PA1003 *(mvfR*) | 42% |
|  |  |  |  |  |  |  | 2558437..2558431 |  | 58% |
| NO_R.5 | 1683961..1720917 | LESB58 | 328 | 1.05 | MP38-like phage |  | 3843332..3843338 |  |  |
| NO_R.6 | 788854..797747 | PAO1 | 661 | 2.08 | Pf4 (normal + extra copy) |  | 785512..785338 |  |  |
|  | 789150..795786 | PAO1 | 933 | 2.94 | Pf4 (normal + extra copy) +  MPAO1 phage-like insertion RGP42 genomic sequence (as in ancestor) | 65% 35% |  |  |  |
|  | 1683961..1720917 | LESB58 | 406 | 1.28 | MP38-like phage | 31% | 4720187..4720182 |  | 13% |
|  |  |  |  |  | LESB58 prophage 4 | 69% | 5455225..5455219 |  | 87% |
| R_1.1 | 1683961..1720917 | LESB58 | 378 | 1.12 | LESB58 prophage 4 |  | 1078783..1078789 | PA0996 (*pqsA*) |  |
| R_1.3 | 1683961..1720917 | LESB58 | 319 | 1.06 | LESB58 prophage 4 |  | 2077076..2077082 | PA1906 |  |
| R_1.5 | 788854..797747 | PAO1 | 5964 | 20.71 | Pf4 (normal + extra copies) |  | 785512..785338 |  |  |
|  | 789150..795786 | PAO1 | 6165 | 21.40 | Pf4 (normal + extra copies) +  MPAO1 phage-like insertion RGP42 genomic sequence (as in ancestor) | 95% 5% |  |  |  |
|  | 1683961..1720917 | LESB58 | 746 | 2.59 | LESB58 prophage 4 |  | 1079232..1079237 | PA0996 (*pqsA*) | 87% |
|  |  |  |  |  |  |  | 2863921..2863928 | PA2535 | 13% |

**Supplementary table 3. Model output of the linear mixed model for changes in relative fitness**

| Model: lme(I(relative density-1) ~ evolution treatment*assay treatment, random=~1\|plate/strain) | | | | | |
| --- | --- | --- | --- | --- | --- |
|  |  |  |  |  |  |
| **ANOVA** | **numDF** | **denDF** | **F-value** | **p-value** |  |
| (Intercept) | 1 | 361 | 103.8276 | <.0001 |  |
| evolution treatment | 3 | 8 | 0.61674 | 0.6233 |  |
| assay treatment | 3 | 361 | 45.96988 | <.0001 |  |
| evolution treatment:assay treatment | 9 | 361 | 5.03275 | <.0001 |  |
|  |  |  |  |  |  |
| **Fixed effects** | **Value** | **Std.Error** | **DF** | **t-value** | **p-value** |
| (Intercept) | 0.025008 | 0.007714 | 361 | 3.241728 | 0.0013 |
| evo_recipient1 | -0.02102 | 0.010949 | 8 | -1.92001 | 0.0911 |
| evo_recipient2 | -0.02003 | 0.010949 | 8 | -1.82937 | 0.1047 |
| evo_recipient3 | -0.0132 | 0.011198 | 8 | -1.17838 | 0.2725 |
| assay_recipient1 | 0.009812 | 0.006464 | 361 | 1.517969 | 0.1299 |
| assay_recipient2 | 0.024945 | 0.006464 | 361 | 3.85915 | 0.0001 |
| assay_recipient3 | 0.031748 | 0.006464 | 361 | 4.911653 | 0 |
| evo_recipient1:assay_recipient1 | 0.036903 | 0.009188 | 361 | 4.016471 | 0.0001 |
| evo_recipient2:assay_recipient1 | 0.020445 | 0.009188 | 361 | 2.22513 | 0.0267 |
| evo_recipient3:assay_recipient1 | 0.013069 | 0.009701 | 361 | 1.347163 | 0.1788 |
| evo_recipient1:assay_recipient2 | 0.006517 | 0.009236 | 361 | 0.705619 | 0.4809 |
| evo_recipient2:assay_recipient2 | 0.022616 | 0.009188 | 361 | 2.461447 | 0.0143 |
| evo_recipient3:assay_recipient2 | 0.021271 | 0.009701 | 361 | 2.192594 | 0.029 |
| evo_recipient1:assay_recipient3 | -0.00639 | 0.009188 | 361 | -0.69587 | 0.487 |
| evo_recipient2:assay_recipient3 | 0.005042 | 0.009188 | 361 | 0.548775 | 0.5835 |
| evo_recipient3:assay_recipient3 | -0.01651 | 0.009701 | 361 | -1.70196 | 0.0896 |

**Supplementary table 4. Model output of the linear mixed model for changes in relative inhibition**

| Model: lme(I(relative inhibition-1) ~ evolution treatment*assay treatment, random=~1\|plate/strain) | | | | | |
| --- | --- | --- | --- | --- | --- |
|  |  |  |  |  |  |
| **ANOVA** | **numDF** | **denDF** | **F-value** | **p-value** |  |
| (Intercept) | 1 | 272 | 10.25263 | 0.0015 |  |
| evolution treatment | 3 | 8 | 6.55456 | 0.0151 |  |
| assay treatment | 2 | 272 | 37.30047 | <.0001 |  |
| evolution treatment:assay treatment | 6 | 272 | 24.27537 | <.0001 |  |
|  |  |  |  |  |  |
| **Fixed effects** | **Value** | **Std.Error** | **DF** | **t-value** | **p-value** |
| (Intercept) | -0.29119 | 0.060775 | 272 | -4.79127 | 0 |
| evo_recipient1 | 0.440613 | 0.085948 | 8 | 5.126484 | 0.0009 |
| evo_recipient2 | 0.031929 | 0.085948 | 8 | 0.371484 | 0.7199 |
| evo_recipient3 | 0.164033 | 0.088594 | 8 | 1.851518 | 0.1012 |
| assay_recipient2 | 0.045156 | 0.020894 | 272 | 2.161189 | 0.0316 |
| assay_recipient3 | 0.041507 | 0.020894 | 272 | 1.986548 | 0.048 |
| evo_recipient1:assay_recipient2 | -0.04709 | 0.029549 | 272 | -1.5938 | 0.1121 |
| evo_recipient2:assay_recipient2 | 0.192939 | 0.029549 | 272 | 6.529545 | 0 |
| evo_recipient3:assay_recipient2 | 0.019499 | 0.030458 | 272 | 0.640198 | 0.5226 |
| evo_recipient1:assay_recipient3 | -0.16633 | 0.029549 | 272 | -5.62887 | 0 |
| evo_recipient2:assay_recipient3 | 0.150064 | 0.029549 | 272 | 5.078535 | 0 |
| evo_recipient3:assay_recipient3 | -0.07892 | 0.030458 | 272 | -2.59102 | 0.0101 |

**Supplementary table 5. Model output of the linear mixed model for the effect of genetic similarity**

| Model: lme(I(relative inhibition-1) ~ genetic similarity + I(genetic similarity^2), random=~1\|plate/strain/assay treatment) | | | | | |
| --- | --- | --- | --- | --- | --- |
|  |  |  |  |  |  |
| **ANOVA** | **numDF** | **denDF** | **F-value** | **p-value** |  |
| (Intercept) | 1 | 210 | 9.275007 | 0.0026 |  |
| genetic smilarity | 1 | 9 | 6.842704 | 0.028 |  |
| I(genetic similarity^2) | 1 | 9 | 7.624886 | 0.0221 |  |
|  |  |  |  |  |  |
| **Fixed effects** | **Value** | **Std.Error** | **DF** | **t-value** | **p-value** |
| (Intercept) | -0.26124 | 0.062594 | 210 | -4.17356 | 0 |
| genetic similarity | 1.860758 | 0.590283 | 9 | 3.152317 | 0.0117 |
| I(genetic similarity^2) | -2.09294 | 0.757949 | 9 | -2.76132 | 0.0221 |
